# Health information literacy among children with spinal muscular atrophy and their caregivers

**DOI:** 10.1186/s13052-024-01723-9

**Published:** 2024-08-26

**Authors:** Weiran Zhang, Yijie Feng, Yue Yan, Mei Yao, Feng Gao, Wei Lin, Shanshan Mao

**Affiliations:** 1 Department of Neurology, Children’s Hospital, Zhejiang University School of Medicine, National Clinical Research Center for Child Health, Hangzhou, 310052 Zhejiang China; 2 Department of Infection, Children’s Hospital, Zhejiang University School of Medicine, National Clinical Research Center for Child Health, Hangzhou, 310052 Zhejiang China; 3 Film and New Media Studies, College of Media and International Culture, Institute of Leisure Studies and Philosophy of Art, Zhejiang University, Hangzhou, 310000 Zhejiang China

**Keywords:** Spinal muscular atrophy, Health information literacy, China, Children

## Abstract

**Background:**

Spinal muscular atrophy (SMA) is an autosomal recessive motor neuron disease that leads to multiple organ dysfunction. The advent of disease-modifying treatments makes the early diagnosis of SMA critical. Health information literacy is vital for obtaining, understanding, screening, and using health information. Considering the importance of early diagnosis and the challenges in obtaining accurate information on patients with SMA, this cross-sectional study assessed health information literacy among children with SMA and their caregivers in China.

**Methods:**

Interviews with the caregivers of 10 patients with SMA were conducted by neurologists specializing in SMA. A questionnaire for evaluating the level of health information literacy was further developed among 145 children with SMA aged 10.0–120.0 months, with the average age of 81.9 months, and their caregivers. Parameters, such as the age at the onset of the first symptom and time from recognition of the first symptom to diagnosis, were examined. Health information literacy was measured using four dimensions: cognition, search, evaluation, and application.

**Results:**

The average time from the first symptom to first medical consultation was 4.8 months, and that from the first symptom to diagnosis was 10.8 months. There is a significant delay from the onset of the initial symptoms to a definitive diagnosis. Thirty-five (24%) patients had poor while 26 (18%) had high health information literacy. The overall score for health information literacy was 69; the scores for health information cognition and application were 90 and 84, respectively. The scores for evaluation (61) and search (57) were low. Medical personnel were considered the most professional and credible sources of information. Additionally, search engines and patient organizations were the other two most important sources of health literacy.

**Conclusion:**

Patients with SMA and their caregivers had low levels of health information literacy. SMA information visibility and standardization need to be improved. Medical personnel with experience in the diagnosis and treatment of SMA and media should aim to share knowledge and increase the quality of life of those with SMA.

**Supplementary Information:**

The online version contains supplementary material available at 10.1186/s13052-024-01723-9.

## Background

Spinal muscular atrophy (SMA) is a severe neurodegenerative condition caused by recessive mutations in the survival motor neuron (SMN) 1 gene resulting in insufficient SMN proteins. It is characterized by progressive muscle weakness and atrophy [1] and multisystem physical dysfunction, which significantly reduce patients’ quality of life [2, 3]. As with other rare disorders, patients with SMA often experience a significant delay in diagnosis [4]. Despite the typical clinical features and ease of performing genetic analyses, a recent review of the diagnostic process for SMA reported problems and frequent delays between the onset of clinical signs and diagnosis for all types of SMA [5]. However, unlike for many other orphan diseases, effective disease-modifying treatments with the representative approved drugs, such as nusinersen and risdiplam, are now available for SMA in China [6]. Early treatment results in greater clinical benefits [7]. Thus, the advent of new therapies has increased the need for early SMA diagnosis to finally improve patient prognosis [8].

Patients affected by rare diseases are dispersed worldwide and face a variety of challenges, including lack of specialized care, delays in diagnosis, negative social consequences, and other psychosocial burdens [9]. Advances in information technology and communication are creating cultural shifts and changing how people develop expertise. These trends have important implications for healthcare systems, being particularly relevant for empowering patients dealing with rare diseases. Using technology to access information and connect with other patients online could help the patient population overcome such challenges [10].

Health literacy is the ability to access, understand, evaluate, and use essential health information to make basic health decisions [11], which is considered as one of the greatest determinants of health [12]. A study conducted by the USA Center for Health Care Strategies indicated that people with limited health literacy are less likely to understand the written and verbal information provided by health experts and thus have poor health conditions [13]. Health information literacy emphasizes a range of information abilities, including recognizing health information needs; identifying possible sources of information and using them to retrieve relevant information; assessing the quality and application of information in a specific situation; and analyzing, understanding, and using information to make scientific health decisions. Health information literacy combines health and information literacy and emphasizes the human ability to discover and use health-related information [14]. Amid the current information revolution, a lack of information is rather unlikely; nonetheless, this is often a reality for people with rare orphan diseases [15]. At present, public health information literacy surveys primarily focus on common chronic diseases, such as diabetes and hypertension; however, surveys on the health information literacy of patients with rare diseases, including SMA, are lacking [16]. A study on the health literacy and self-efficacy levels of parents of patients with SMA showed that the parents had low health literacy and self-efficacy levels. A positive significant relationship exists between health literacy and self-efficacy [17]. As China is a developing country with a relatively large number of patients with SMA, both awareness and understanding of this disease among the medical personnel and patient population as well as its diagnosis and treatment levels lag behind the international standards. Before the availability of disease-modifying treatment, patients with SMA in China faced the dilemma of delayed diagnosis and lack of treatment. However, with increased accessibility of nusinersen and risdiplam, the patient population’s health information literacy is of great significance for their follow-up prognosis. Therefore, this study examines the health information literacy of patients with SMA in China to address the research gap in related fields, promote early diagnosis of SMA, and identify effective approaches for the survival of patients with SMA.

## Methods

### Study design

Two-stage cross-sectional study was carried out with the qualitative interviews on the caregivers of patients with SMA and followed by a quantitative questionnaire survey administered to children with SMA and their caregivers. This study was approved by the Medical Ethics Committee of the Children’s Hospital of Zhejiang University School of Medicine (2022-IRB-199). Patients and their caregivers were recruited from December 2021 to December 2023 through the outpatient clinic of the Children’s Hospital of Zhejiang University School of Medicine and written informed consent were obtained from all patients and/or their caregivers before data collection.

### Interview

Considering the minimum sample size requirement of a qualitative study and the availability of prospective patients, we recruited caregivers of 10 patients with SMA to participate for the interview. To achieve better representativeness, the eligiable criteria for recruitment were wide (Table 1). The interviews were conducted by two trained neurologists specializing in SMA according to a prespecied frame and keypoints (Table 1), one responsible for communicating with the participants and the other for taking real-time notes. A supplementing interview was conducted via a telephone or face-to-face communication when necessary.

**Table 1:** Inclusion criteria and key points of the interview with caregivers and patients with spinal muscular atrophy (SMA)

**Inclusion criteria**	
Gender	Both male and female patients
Age	Pre-school, primary, middle, and high school, university, and working patients
Disease type	Patients with types I–IV SMA
Disease duration	Patients whose disease duration was less or more than 1 year
Whether they are members of a patient organization	Patients or patients’ family members who have joined or not joined a patient organization
**Key points of the interview**	
Before diagnosis	1. Initial symptoms of disease onset, information on the first medical details, including the visit time, hospital, department, and initial causes
	2. Referral status and reasons
	3. Understanding of SMA
At diagnosis	1. Inspection and diagnosis process
	2. Doctor communication situation
	3. Treatment situation and the causes
	4. Further understanding of SMA
	5. Proactive searching for SMA-related information
	6. Patient organization joining status
	7. Information channels
	8. Specific information content and form of each channel
	9. Unmet needs and expectations
After diagnosis	1. Treatment situation and the causes
	2. Daily care situation
	3. Reception and active search of SMA-related information
	4. Information channels
	5. Specific information content and form of each channel
	6. Unmet needs and expectations

### Questionnaire survey

#### Development of the questionnaire

Based on the data from the interview, a structured questionnaire drafted for the cross-sectional survey was developed by the research directors and sent to the caregivers of those 10 patients for small-scale test to evaluate the feasibility and effectiveness. After making modifications, the questionnaire was then revised and confirmed by several medical experts from different department specializing in SMA (Supplementary Table 1). The online questionnaire was designed by programming professionals, which passed the test before the final application.

The questionnaire consisted of three parts: (1) demographic and background information, including age, gender, educational level, SMA type, disease duration, family income, and Insurance status; (2) the interval between symptom onset and first hospital visit, mean time between symptom onset and diagnosis, and types of medical investigations conducted for diagnosis; and (3) health literacy measuring four main dimensions, namely recognizing health information needs, retrieving relevant information, assessing the quality of information, and applying information. Responses were rated on a five-point Likert scale (1 = no knowledge; 5 = advanced knowledge). Finally, the total score was calculated by aggregating the scores of all dimensions (ranging from 0 to 100) and dividing them by the number of dimensions.

#### Carry-out of the questionnaire investigation

Patients were eligible if one with a diagnosis of 5qSMA by genetic testing and were able to clearly express their views and thoughts. To minimize bias, participants whose family members worked in advertising, magazines, television stations, or other media industries; pharmacies or hospitals; or drug production or sales-related industries were excluded.

After obtaining inform consent, the link of the online questionnaire was distributed to SMA patients and their caregivers. All the finished questionnaires were collected within one week.

### Data analysis

SPSS analytical software, version 22 (IBM Corporation, Armonk, NY, USA), was used for the statistical data analysis. Categorical variables were expressed as frequencies and percentages and the mean was used to describe the continuous variables for the quatitative data analysis. The impact of different demographic data on the scores of the four aspects of health literacy was analyzed through a Univariate and Multivariate analysis.

## Results

Ten patients with SMA and their family caregivers participated in the interview, including one with type I SMA, six with type II SMA, and three with type III SMA. These patients were 1.8 to 9.5 years old, with a disease duration ranging from 0.5 to 7 years. The interviews indicated that 6 patients’ family caregivers were active in accessing information and thus had relatively high health literacy, while the other 4 were passive with a relatively low health literacy. Moreover, the interviews showed that medical personnel are the most professional and credible sources of information for these patients with SMA and their families.

For the questionnaire survey, a total of 166 questionnaires were collected; 145 valid questionnaires were included in the final analysis, comprising patients from 24 Chinese provinces. Participants’ characteristics are summarized in Table 2. The patients were aged 10.0–120.0 months, with the average age of 81.9 months (6.8 years), and 70% of them had a disease course of more than 2 years. In terms of clinical classification, most participants had type II SMA. The basic conditions of patients with type I SMA differed significantly from those of patients with types II and III SMA who were characterized by younger age, shorter disease duration, and poorer medical insurance. Of the participants, 97% were family members of patients, most of whom were women aged approximately 35 years. They had a relatively high level of education, with 47% holding college degrees or above.

**Table 2:** Demographic characteristics of caregivers and patients with spinal muscular atrophy (SMA)

**Characteristics of caregivers (** ***N*** ** = 145)**		***n*** **(%)**
Caregivers	Parents	140 (97)
	Non-parents	5 (3)
Gender	Male	40 (28)
	Female	105 (72)
Age (years)	< 30	17 (12)
	30–45	96 (66)
	45–50	30 (21)
	≥ 50	2 (1)
Annual family income (thousand RMB)	< 100	87 (60)
	100–200	42 (29)
	200–300	9 (6)
	> 300	7 (5)
Educational level	Below high school diploma	39 (27)
	High school diploma	38 (26)
	College degree or above	68 (47)
**Characteristics of patients (*N*** **= 145)**		***n*** **(%)**
Age (years)	< 2	19 (13)
	2–5	43 (30)
	5–12	66 (46)
	≥ 12	17 (12)
SMA type	I	20 (14)
	II	86 (59)
	III	39 (27)
Disease duration	< 12 months	19 (13)
	12–24 months	25 (17)
	24–60 months	43 (30)
	≥ 60 months	58 (40)
Insurance status	Social insurance	104 (72)
	Business insurance	28 (19)
	None	33 (23)

The characteristics of the caregivers of patients with SMA before diagnosis are presented in Table 3. The average score of the participants’ awareness of motor milestones was 51, with nearly one-third being completely unaware of the need to pay attention to their children’s motor milestones at different growth stages. The mean time from the first symptom onset to first hospital visit was 4.8 months, including 1.5 months for patients with type I SMA, 3.9 months for patients with type II SMA, and up to 8.3 months for patients with type III SMA. For family members of patients with SMA with motor milestone awareness scores < 60, the average interval from symptom onset to medical consultation was 4.1 months, and for those with scores > 60, it was 2.8 months. When patients had symptoms that were not yet diagnosed, 39% did not conduct any information inquiry. Participants with awareness of information reported having limited information channels, primarily Internet search engines. The most prominent feature of this stage was the low cognition of information, which was an obstacle to information inquiry.

**Table 3:** Characteristics of patients with spinal muscular atrophy (SMA) and their caregivers before diagnosis

Characteristics of SMA patients and their caregivers	
**Awareness of motor milestones for children aged 0–6 years** (*N* = 145, mean)	**n (%)**
No knowledge (1)	42(29%)
Little knowledge (2)	29(20%)
Some knowledge (3)	44(30%)
Good knowledge (4)	14(10%)
Advanced knowledge (5)	16(11%)
**Interval between the first symptom and first consultation** (*N* = 145, mean)	**Months**
All patients	4.8
Type I SMA (*N* = 20)	1.5
Type II SMA (*N* = 86)	3.9
Type III SMA (*N* = 39)	8.3
**Awareness of motor milestones affect the first consultation for types I and II SMA** (*N* = 106, mean)	
Awareness of motor milestones for children aged 0–6 years	n
Score < 60	53
Score ≥ 60	53
Interval between the first symptom and first consultation	Months
Score < 60	4.1
Score ≥ 60	2.8
**Interval between the onset of clinical signs and diagnosis** (*N* = 145, mean)	**Months**
All patients	10.8
Patients from provincial capital city (*N* = 36)	7.0
Patients from non-provincial capital city (*N* = 108)	12.0
By SMA type	
Type I (*N* = 20)	3.8
Type II (*N* = 86)	8.6
Type III (*N* = 39)	19.0
**Number of doctors visited** (*N* = 145, mean)	**N**
All patients	3.1
Patients from provincial capital city (*N* = 36)	2.9
Patients from non-provincial capital city (*N* = 108)	3.2
By SMA type	
Type I (*N* = 20)	3.4
Type II (*N* = 86)	2.9
Type III (*N* = 39)	3.4

The mean time from the first consultation to diagnosis was 6 months and the average number of referrals was 3.1 months. Almost all patients sought treatment from the wrong department, such as rehabilitation, growth, and development, leading to different degrees of misdiagnosis. New media platforms, such as short videos, are intuitive and play an increasingly important role in helping some patients shorten their diagnosis time. The average time from symptom onset to diagnosis was 7 months and the average number of referrals was 2.9 for patients from provincial capital cities. Conversely, the average time from symptom onset to diagnosis was 12 months, and the average number of referrals was 3.2 for patients from non-provincial capital cities. For patients with good health literacy, the mean time from the first consultation to diagnosis was 3.4 months, and the average time from the first consultation to diagnosis was 3.3 months.

Health literacy among the caregivers of patients with SMA after diagnosis is summarized in Tables 4 and 5. The cognition of health information of patients with SMA and their family members improved after diagnosis. However, information channels remained singular. Many patients and their family members expressed difficulties with information queries, screening, and evaluation, and most joined patient groups. Rehabilitation was the main treatment for patients with SMA, and only 23% received disease-modifying therapy. Approximately one-third of the patients did not receive any treatment.

**Table 4:** Health information literacy of patients with spinal muscular atrophy (SMA) and their caregivers after diagnosis (*N* = 145, mean)

Health literacy variables	Score
**Health information cognition** (average score: 90)	
It is very important to be informed about SMA.	92
I hope to get SMA-related information from a variety of sources.	93
I can express what SMA health information I need.	84
**Health information search** (average score: 57)	
I know where to get SMA-related health information.	81
It is difficult to obtain SMA-related health information from print media.	45
It is difficult to obtain SMA-related health information from new media.	54
It is difficult to obtain SMA-related health information from radio and television.	49
**Health information evaluation** (average score: 61)	
It is easy to evaluate the reliability of SMA-related health information in print media.	62
It is easy to evaluate the reliability of SMA-related health information in new media.	66
SMA-related health information is often difficult to understand.	53
I do not know whom to trust when talking to others about SMA-related issues.	60
It is easy to evaluate the reliability of SMA-related health information on radio and television.	62
**Health information application** (average score: 84)	
I will apply the SMA-related health information I have obtained for my benefit and the benefit of those around me.	81
I am willing to share what I know about SMA health information with others.	88

**Table 5:** Health information literacy scores of patients with spinal muscular atrophy (SMA; *N* = 145, mean)

Health literacy variables	Total	Score < 65	Score65–75	Score > 75
	(*N* = 145)	(*N* = 35, 24%)	(*N* = 84, 58%)	(*N* = 26, 18%)
Awareness of motor milestones for children aged 0–6 years	51	43	52	58
Interval between the onset of clinical signs and the first consultation in months	4.8	4.4	5.3	3.4
Interval between the onset of clinical signs and diagnosis in months	6.0	8.9	5.6	3.3
Number of doctors visited	3.1	3.6	2.9	3.2
Current treatment status (%)				
Rehabilitation	47	46	46	50
Nusinersen	18	17	19	15
Salbutamol	10	11	10	12
Traditional Chinese medicine	9	9	8	12
Other	6	6	4	12
Risdiplam	5	0	5	12
None	32	34	35	19

Most participants had low health information literacy, with an average score of 69. The two aspects with high scores were health information cognition and application, with scores of 90 and 84, respectively. The abilities to evaluate (61) and search for health information (57) were low. As the respondents were mostly from organizations for patients with SMA, the average level of education was high. With the introduction of the world’s first SMA disease-modifying drug into medical insurance and drug accessibility improvement, more patients with lower educational levels can seek treatment in hospitals. Therefore, the actual health information literacy level of patients with SMA and their families may be lower than the results of this survey.

The score for health information cognition was 90, the highest among the four dimensions of health information literacy. Respondents’ information needs for new drug research and development, medical insurance policies, multidisciplinary management, rehabilitation nursing, and other aspects were constantly changing and updated. The number of channels mastered by the respondents increased to 3.8. However, health information search literacy remained the lowest, with a score of 57. Although medical staff were considered the most professional and reliable sources of SMA information, patient organizations (67%) and search engines (52%) were the two most frequently used sources. Participants were concerned about various aspects of treatment, including drug development (89%), rehabilitation methods (82%), institutions (68%), medical costs (81%), and welfare policies (76%).

The average score for health information evaluation was 61, indicating that the participants found it difficult to judge information reliability (62 for print media, 66 for new media, 62 for radio and television), understand relevant professional terms (53), and judge the credibility of information sources (60).

Health information application scored 84, indicating a strong desire to share information (88 points). Additionally, the ability to apply the acquired health information in practice improved (81). Multidisciplinary, whole-course management and the daily care of patients with SMA are closely related to their quality of life, are relatively complex, and rely on the collaboration of multiple disciplines, such as neurology, orthopedics, respiratory, rehabilitation, nutrition, acute care, and home care. Respiratory management is integral for many patients with type I SMA. Most patients with types II and III SMA face nutrition and dietary problems. The information barriers they face are prominent, such as worrying about the quality of information (61%), information being difficult to understand (42%), the search for information requiring significant energy (34%), and feeling frustrated when searching for information (34%). In addition, the normal demographic data had no significant impact on the scores of the four aspects of health literacy: cognition, search, evaluation, and application through further analysis (all *P* > 0.05). Supplementary Fig. 1 presents the detailed evaluation items of the four aspects of health information literacy.

## Discussion

To our best knowledge, this study was the first to consider the health information literacy of patients with SMA, and it revealed that there exists a diagnostic delay for SMA and poor health literacy among patients with SMA and their caregivers. The interview results of 10 patients with SMA and their caregivers suggested that they had experienced varying degrees of diagnostic delays and had to travel to multiple hospitals before finally being diagnosed with SMA. Before diagnosis, patients’ caregivers did not know which channels to use to learn about the disease, while after diagnosis, they joined a patient organization and learned about SMA through social media and tweets, among others. Before the availability of disease-modifying treatment drugs for SMA, parents and caregivers did not pay much attention to the disease. When drugs became available, they learnt to use various social media and tools to acquire more diagnosis and treatment information.

Studies showed a significant delay from the onset of the initial symptoms to a definitive diagnosis [18]. In our study, the mean time from the first symptom onset to first hospital visit was long, especially for patients with type III SMA. Early diagnosis is critical to improve patient prognosis. The time window for SMA diagnosis can be shortened if parents and caregivers become aware of the early signs of abnormal growth and development in neonates through scientific popularization or a life-cycle health detection system [4]. Furthermore, during the stage of symptom onset and seeking medical treatment, respondents with high awareness of motor milestones in children aged 0–6 years were more likely to seek medical treatment and had a shorter time to diagnosis. Moreover, an Internet-based registry platform and collaboration networks could be established and neonatal and carrier screening for SMA could be performed to reduce the diagnostic time window.

Health information literacy affects people’s ability to accurately search for and use health information and adopt healthier behaviors. Low health literacy has become a global public health concern. At the beginning of the 21st century, the USA implemented health information literacy education programs [19]. The first European survey on health literacy found that 47% of the population in eight European countries had low health literacy [20]. Research in Isfahan showed that the average health literacy score of participants was poor or marginal, and therefore they required more explanation from medical staff to understand and implement the health and medical instructions [21]. In China, health literacy has been receiving attention from scholars since 2012, and survey results show that approximately 91.2% of Chinese residents have low health literacy [22]. Based on the literature review, only one study carried out in Turkey initially explored the health literacy of the SMA population [17]. Our study based on their findings have further investigated the health information literacy details from multiple dimensions, and provided the potential approaches to improve the health information literacy of the population with SMA. According to our results, the overall level of health information literacy of patients with SMA and their families was low. They had high level of health information literacy in terms of cognition and application, but low levels for search and evaluation. The interview findings indicated that the low levels of health information literacy for search and evaluation skills may be because patients and their family members rely too much on the patient organizations after joining them to obtain information, leading to passive reception, lack of context, half understanding, knowledge without use, and so on. Besides, credibility of the information accessed through search engines and its evaluation is debatable. Nevertheless, the health information literacy of patients with SMA and their family members improved significantly from the onset of symptoms to diagnosis, treatment, and daily disease management. This was primarily reflected by actively seeking information, the number of information sources, awareness of sharing information, and search content. The participants initially focused on symptoms and diseases, subsequently shifting to medication and rehabilitation. However, they paid little attention to the multidisciplinary and whole-course management of SMA, which is crucial for patient prognosis.

In addition to medical personnel, search engines and patient organizations were the two most important information sources. Search engines were mostly used by patients with SMA and their family members for information access and supplementation. Before patients visited doctors, search engines were the first, or even only, method for accessing information among most respondents. After diagnosis, search engines became a supplementary source of information in addition to patient organizations during treatment and daily care. However, the role of search engines was limited, as they could not provide further support, such as information screening, evaluation, and application. After the patients were diagnosed, the importance of patient organizations increased, and their credibility score was second only to that of the government and other authorities. Thus, patient organizations were the most important sources of information for patients after diagnosis. Although patient organizations were powerful supplements for information search and evaluation abilities, their impact on application abilities remains unknown. New media, such as social media, short videos, and new health professional media, play an increasingly important role in the health information literacy of patients with SMA and their family members. Various new media platforms have played a comprehensive role in shortening the medical journey, communication, and rehabilitation guidance for psychological support. Owing to the intuitive presentation of content, short videos may play an important role in guiding patients to associate their symptoms with SMA and encourage them to quickly choose the appropriate hospital and department for medical treatment. In the diagnosis, treatment, and daily care stages, social media is the most widely and frequently used new media platform for information dissemination and activities among patients with SMA. Owing to the low production cost and intuitive characteristics of short videos, many patients and their families communicate on short video platforms, exchange information on their conditions, and obtain and provide psychological support. New information media has also become the first reliable source for patients to obtain information about new SMA drug research and medical insurance. Given their high credibility, new health media are important information evaluation tools throughout the course of the disease. Although infrequently used, new health media play an important role in the cross-identification and evaluation of other information sources. Most patients with SMA and their family members are young, so they are comfortable using all types of new media. Therefore, new media played an indispensable role in the daily information acquisition of the respondents.

To improve health information literacy among patients with SMA and their caregivers, several potential interventions and programs could be considered. First, the government, industry, universities, and researchers should jointly build a series of SMA disease databases and mechanisms to improve the visibility and standardization of SMA disease information. Besides, they should establish a national or regional SMA science popularization expert and resource database; build an all-media SMA science popularization knowledge release and dissemination mechanism; create a fair, just, authoritative, and standardized SMA information platform; and provide patients with one-stop solutions, including information on the disease, medical treatment, medication, and rehabilitation. Second, there is a need to adopt one content crowdfunding mechanism to expand the quantity, improve the quality, and reduce the barriers to patient information. Third, more medical personnel and media workers should be encouraged to participate in public education. By fully mobilizing the attention of all sectors of the society to rare diseases such as SMA, we can take various measures to improve the health information literacy of patients and caregivers.

Additionally, the study had limitations. The patients were sampled only from organizations for patients with SMA, and their average level of education was relatively high. Therefore, the results may not be representative of the actual health information literacy among patients with SMA in China. Additionally, we did not conduct an in-depth analysis; therefore, future research is required to analyze each SMA type.

To sum up, our study creatively explored the current health information literacy status in the population with SMA and revealed patients and their caregivers’ poor and low abilities of health information search and evaluation, stressing the importance of improving health information literacy and calling for medical personnel with experience in the diagnosis and treatment of SMA and media to share knowledge and increase the quality of life of patients with SMA; the same also applies to other rare diseases.

## Conclusions

The health information literacy of patients with SMA and their caregivers in China is poor, and low abilities of health information search and evaluation are prominent. Increasing the degree of disease cognition in patients is helpful in improving medication compliance to enhance treatment effects. In addition to medical staff, search engines and patient organizations were found to be the most important information channels, with new media playing an increasingly important role. In the future, for SMA and other similar rare diseases, disease information visibility and standardization should be improved. We can use the crowdfunding mechanism to expand the quantity and quality of disease content, so as to reduce the information barriers for patients. Medical personnel with experience in disease diagnosis and treatment as well as media are called to share knowledge and increase the quality of life of patients with rare diseases.

### Electronic supplementary material

Below is the link to the electronic supplementary material.

Supplementary Material 1: Figure 1. Detailed evaluation items of the four aspects in health information literacy

Supplementary Material 2

## Data Availability

Data that support the findings of this study are available from the corresponding author upon reasonable request.

## Abbreviations

SMA: spinal muscular atrophy
SMN: survival motor neuron
