# Supplementary material for: Health information literacy among children with spinal muscular atrophy and their caregivers
Source: Ital J Pediatr. 2024 Aug 26;50:157. doi: 10.1186/s13052-024-01723-9 (PMC11346139; doi:10.1186/s13052-024-01723-9)

Channels for learning SMA related information during the treatment and daily management stages in addition to professional medical personnel (N=145)

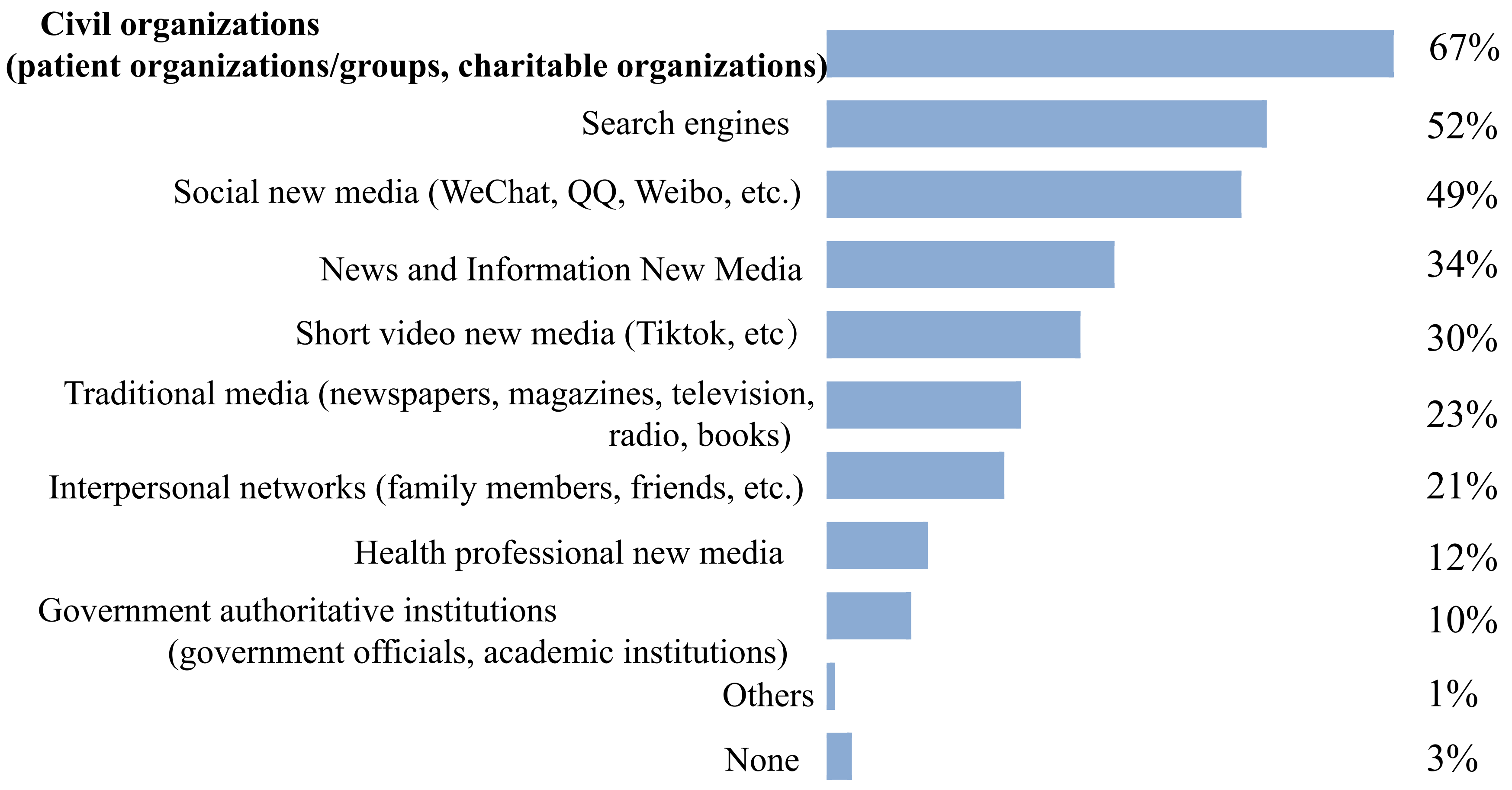

Main information queried during the treatment and daily management stages (N=141)

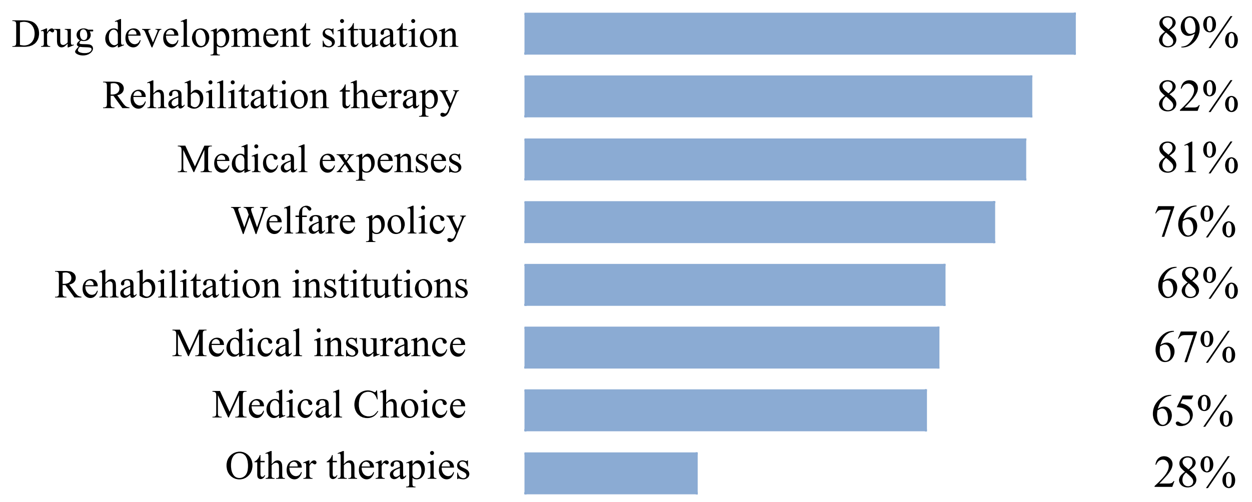

Channel professionalism evaluation (N=145)

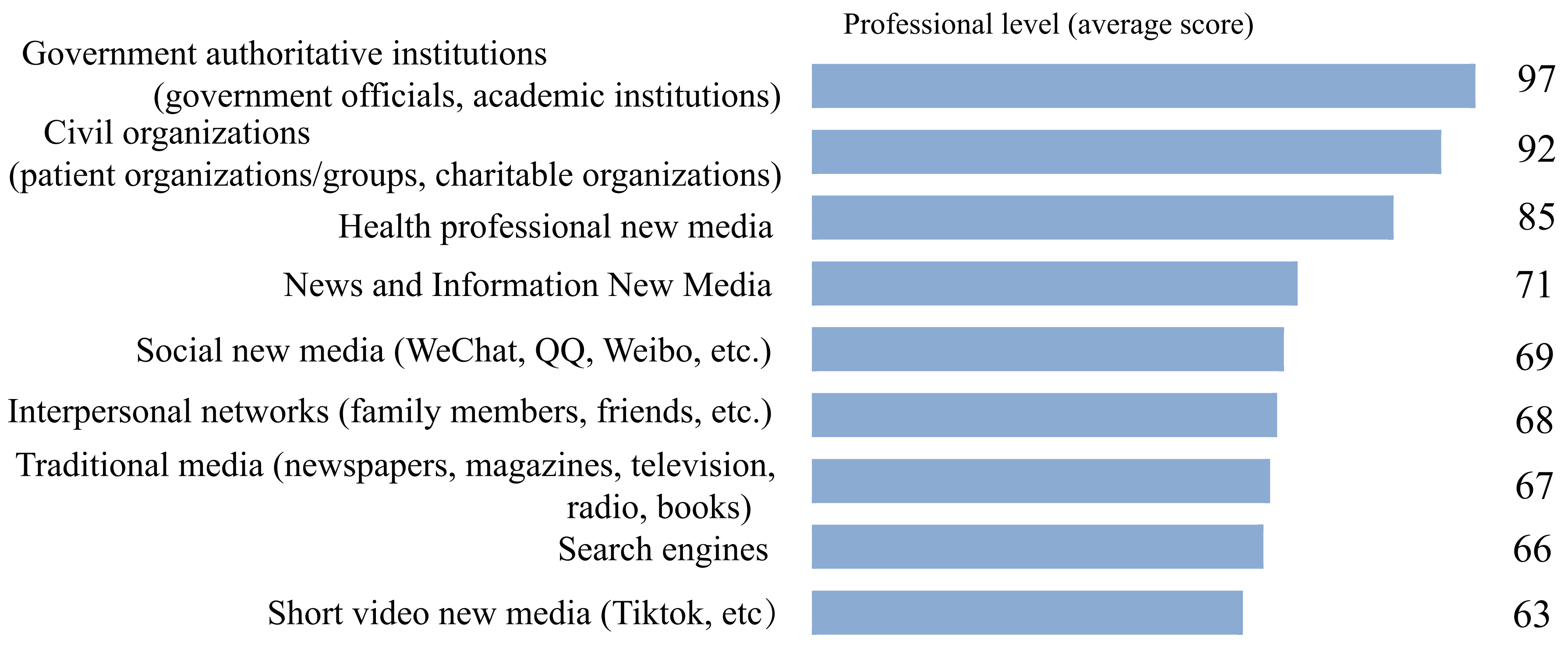

Obstacles encountered in obtaining SMA related information (N=145)

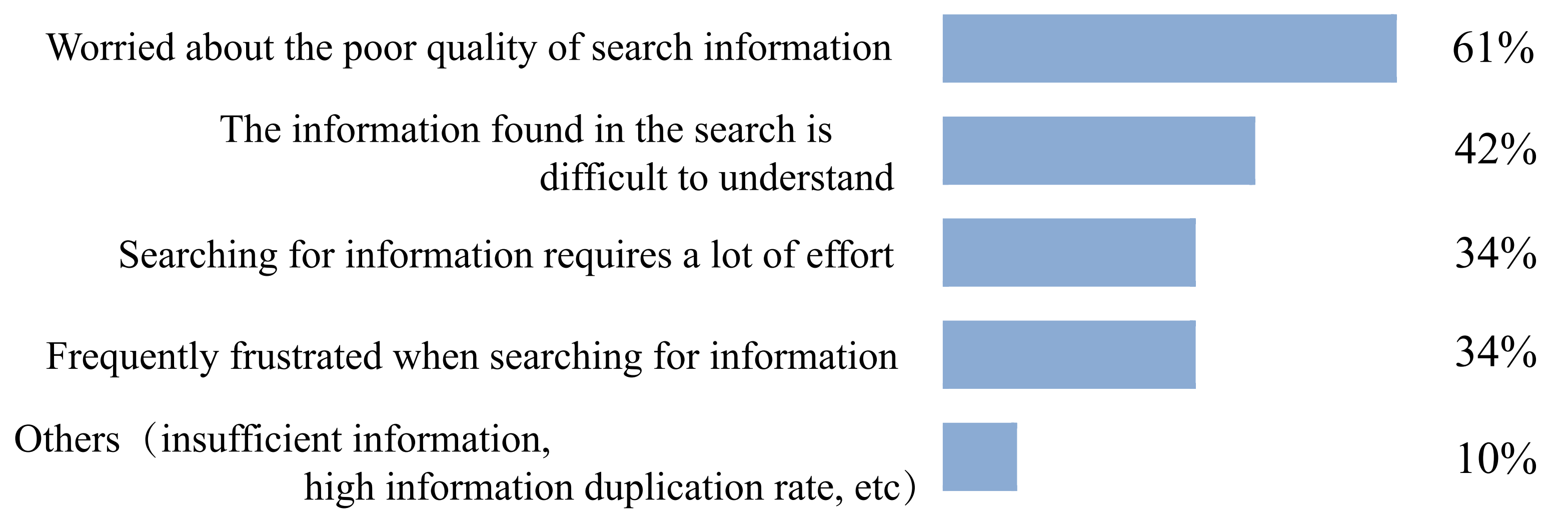

Supplement: Supplementary file 1 — Supplementary Material 1: Figure 1. Detailed evaluation items of the four aspects in health information literacy [file 13052_2024_1723_MOESM1_ESM.pdf]
