# Supplementary material for: Health information literacy among children with spinal muscular atrophy and their caregivers
Source: Ital J Pediatr. 2024 Aug 26;50:157. doi: 10.1186/s13052-024-01723-9 (PMC11346139; doi:10.1186/s13052-024-01723-9)
Supplement: Supplementary file 2 — Supplementary Material 2 [file 13052_2024_1723_MOESM2_ESM.docx]

| **Expert**  **numbers** | **Hospital**  **types** | **Departments** | **Professional**  **directions** | **Professional titles** | **Number of SMA patients per year** |
| --- | --- | --- | --- | --- | --- |
| Expert 1 | General hospital | Neurology | Neuromuscular disorders | Associate Chief physician | ≥30 |
| Expert 2 | Children's Hospital | Neurology | Neuromuscular disorders | Chief physician | ≥70 |
| Expert 3 | General Hospital | Neurology | Neuromuscular disorders | Associate Chief physician | ≥20 |
| Expert 4 | Children's Hospital | Pneumology | Pulmonary Rehabilitation | Associate Chief physician | ≥50 |
| Expert 5 | Children's Hospital | Rehabilitation | Clinical Nutrition in Children | Associate Chief physician | ≥50 |

Supplementary Table 1. Information of medical experts participating in evaluating the questionnaire validation.
